# Machine Learning Glove Using Self‐Powered Conductive Superhydrophobic Triboelectric Textile for Gesture Recognition in VR/AR Applications

**DOI:** 10.1002/advs.202000261

**Published:** 2020-06-09

**Authors:** Feng Wen, Zhongda Sun, Tianyiyi He, Qiongfeng Shi, Minglu Zhu, Zixuan Zhang, Lianhui Li, Ting Zhang, Chengkuo Lee

**Affiliations:** ^1^ Department of Electrical & Computer Engineering National University of Singapore 4 Engineering Drive 3 Singapore 117576 Singapore; ^2^ National University of Singapore Suzhou Research Institute (NUSRI) Suzhou Industrial Park Suzhou 215123 China; ^3^ Center for Intelligent Sensors and MEMS National University of Singapore 5 Engineering Drive 1 Singapore 117608 Singapore; ^4^ Hybrid Integrated Flexible Electronic Systems (HIFES) 5 Engineering Drive 1 Singapore 117608 Singapore; ^5^ i‐Lab Suzhou Institute of Nano‐Tech and Nano‐Bionics Chinese Academy of Sciences (CAS) Suzhou 215123 China

**Keywords:** gesture recognition, machine learning, superhydrophobic textiles, triboelectric nanogenerators (TENGs), virtual reality/augmented reality (VR/AR) controls

## Abstract

The rapid progress of Internet of things (IoT) technology raises an imperative demand on human machine interfaces (HMIs) which provide a critical linkage between human and machines. Using a glove as an intuitive and low‐cost HMI can expediently track the motions of human fingers, resulting in a straightforward communication media of human–machine interactions. When combining several triboelectric textile sensors and proper machine learning technique, it has great potential to realize complex gesture recognition with the minimalist‐designed glove for the comprehensive control in both real and virtual space. However, humidity or sweat may negatively affect the triboelectric output as well as the textile itself. Hence, in this work, a facile carbon nanotubes/thermoplastic elastomer (CNTs/TPE) coating approach is investigated in detail to achieve superhydrophobicity of the triboelectric textile for performance improvement. With great energy harvesting and human motion sensing capabilities, the glove using the superhydrophobic textile realizes a low‐cost and self‐powered interface for gesture recognition. By leveraging machine learning technology, various gesture recognition tasks are done in real time by using gestures to achieve highly accurate virtual reality/augmented reality (VR/AR) controls including gun shooting, baseball pitching, and flower arrangement, with minimized effect from sweat during operation.

## Graphical abstract

With capabilities of humidity‐resistant and anti‐sweat, high‐accuracy complicated gesture recognition based on machine learning is realized using a low‐cost and self‐powered superhydrophobic glove human machine interface (HMI) with minimized sweat effect. With gesture recognition, the virtual reality/augmented reality (VR/AR) controls including shooting, baseball pitching, and floral arrangement are successfully demonstrated.

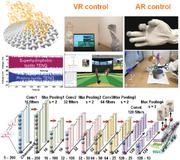

## 1 Introduction

Wearable electronics have drawn tremendous interest from the academic community as revealed by increasing research effort in this area.^[^ ^1^, ^2^, ^3^, ^4^, ^5^, ^6^, ^7^, ^8^, ^9^, ^10^ ^]^ Among them, epidermal electronics (e.g., E‐skin) based on polymer substrate, as an important category of wearable electronics, have been invested with significant effort to develop skin‐like biochemical monitoring patch,^[^ ^11^ ^]^ physical signal sensing (e.g., pressure mapping) system^[^ ^12^, ^13^ ^]^ and miniaturized electronics incorporated epidermal array.^[^ ^14^ ^]^ Besides, many works have been conducted to develop textile‐based wearable electronics by leveraging screen printing,^[^ ^15^ ^]^ ink‐jet printing,^[^ ^16^ ^]^ functionalization^[^ ^17^, ^18^, ^19^, ^20^, ^21^ ^]^ and direct adhesion.^[^ ^22^, ^23^ ^]^ They are used in diversified applications such as healthcare, smart home and human–machine interfaces (HMIs).

The new era of the internet of things (IoT) is emerging owing to artificial intelligence and 5G technologies. Hence, wearable HMIs are experiencing an imperative requirement for human–machine interaction in IoT applications.^[^ ^24^, ^25^, ^26^, ^27^, ^28^, ^29^, ^30^ ^]^ Glove, as a daily used wearable item, fitting human fingers well and matching with the operation logic of the brain, is a perfect carrier for the realization of HMIs.^[^ ^31^, ^32^ ^]^ As a promising material, the textile is widely used for diversified HMI applications due to its unique characteristics of softness, conformability and wearable convenience.^[^ ^33^, ^34^, ^35^, ^36^ ^]^ There have been a few works reported HMIs based on capacitive, resistive and triboelectric mechanisms. C. Boutry et. al.^[^ ^37^ ^]^ designed a capacitive sensor array in pyramid microstructures for robotic hands to discriminate normal and tangential forces. S. Sundaram et al.^[^ ^38^ ^]^ proposed a tactile knitted glove assembled with 548 resistive sensors to achieve the signature recognition of human grasp, showing a great potential of using a glove to realize highly accurate object manipulator. However, complicated system design and data processing may restrict their broadened applications in human machine interactions since they need a large number of sensors to capture comprehensive information for human/robotic hands. In numerous scenarios, glove‐based HMIs can perfectly meet the needs of control even equipped with several sensors, which is so called minimalist design. Accordingly, S. Choi et al.^[^ ^39^ ^]^ proposed an intuitive control interface involving 3 resistive sensors distributed on 3 fingers of the glove to perform VR shooting game, indicating a simple HMI solution. Similarly, K. Suzuki et. al.^[^ ^40^ ^]^ used 9 carbon nanotube (CNT)‐based resistive strain sensors as components of a textile‐based glove for the real‐time finger joint motion detection. Nevertheless, the reported capacitive and resistive glove‐based HMIs require an external power supply to maintain sensor operations and cannot achieve the perception of complex gestures without advanced signal analysis methods such as machine learning. Emerged energy harvesting techniques, such as piezoelectric^[^ ^41^, ^42^, ^43^, ^44^, ^45^ ^]^ and triboelectric,^[^ ^38^, ^46^, ^47^, ^48^, ^49^ ^]^ promote the advancement of power‐compatible and self‐powered wearable HMIs by scavenging ubiquitous mechanical energy from human motions to mitigate battery dependence.^[^ ^50^, ^51^, ^52^ ^]^ Triboelectric nanogenerator (TENG),^[^ ^53^, ^54^, ^55^, ^56^, ^57^, ^58^, ^59^, ^60^, ^61^, ^62^, ^63^, ^64^, ^65^, ^66^ ^]^ owing to its unique advantages of simple working principle, broadened choice of materials, easy fabrication, lightweight and low cost, can be an optimal solution for diversified applications, such as healthcare,^[^ ^67^, ^68^, ^69^, ^70^, ^71^, ^72^ ^]^ sensing,^[^ ^73^, ^74^, ^75^, ^76^ ^]^ and self‐powered wearable HMIs.^[^ ^28^, ^77^, ^78^, ^79^ ^]^ In the past few years, triboelectric glove‐based HMIs become popular to enable expedient detection of human hand gestures leading to intelligent interaction between human and machine. For example, X. Pu et. al.^[^ ^80^ ^]^ developed a joint motion triboelectric quantization sensor to operate robotic hands via human gestures by counting signal peaks. On the other hand, by leveraging the threshold of amplitude, a car control with adjustable speed was realized by using triboelectric glove‐based HMI to measure the finger bending degree.^[^ ^32^ ^]^

Although the amplitude and number of peaks of triboelectric signals are important indexes for triboelectric HMIs to achieve interactive communication between human and machines,^[^ ^81^ ^]^ human motions related triboelectric signals have much subtle information that cannot be distinguished by naked eyes or be simply differentiated using signal amplitude or peak number.^[^ ^82^ ^]^ That is, complex gestures cannot be recognized by only depending on triboelectric amplitude or peak number. The rapid development of HMIs calls for an effective and continuous solution to realize a more comprehensive and complicated signal analysis and recognition. Machine learning from Artificial Intelligence (AI) has a great potential to achieve the functionality through readily analyzing signals from multiple channels. As an emerging technology for extracting subtle differences and processing multichannel signals, machine learning is showing its momentum in delicate signal analysis and accurate recognition.^[^ ^83^ ^]^ Recently, distinct gait characteristics,^[^ ^84^ ^]^ keystroke dynamics,^[^ ^85^, ^86^, ^87^ ^]^ and finger motions^[^ ^88^ ^]^ enable identity recognition or gesture recognition by leveraging machine learning to analyze individual behaviors or habits. For example, Zhong Lin Wang's group^[^ ^89^ ^]^ reported triboelectric keyboard interfaces augmented by machine learning to recognize typing samples of different people. Besides, X. Liang et. al.^[^ ^88^ ^]^ used different types of data sources from resistive sensors and radar to realize intelligent gesture recognition but needing an external power supply. Differentiated from the abovementioned works, low‐cost and self‐powered glove interfaces based on TENG are developed for complex gesture recognition with the help of machine learning. Since finger motions contain tremendous information concerning the triboelectric signals of various gestures, both simple and complex gestures can be recognized as long as appropriate training process is carried out. The combination of machine learning with low‐cost and self‐powered gloves explores a new possibility of a universal platform for the recognition of complex gestures compared with previous works,^[^ ^12^, ^32^, ^37^, ^38^, ^39^, ^40^, ^80^, ^90^, ^91^, ^92^ ^]^ which is indicated in **Table** **1**.

**Table 1:** The comparison of representative glove‐based HMIs

No.	Mechanism	Materials	Preparation method	Measured parameter	Analysis method	Number of sensors	Application	Note
1^[^ ^37^ ^]^	Capacitive	CNT and PU	Lithography and patterning	normal and tangential forces	Amplitude	25 per fingertip	Spatial pressure discrimination	a)
2^[^ ^90^ ^]^	Capacitive	Liquid metal and sillcone rubber	Dispersing	Finger bending degree	Amplitude	1 per finger	Simple grasp object detection	a)
3^[^ ^91^ ^]^	Capacitive	Textile and sillcone elastomer	Molding	Finger bending degree	Amplitude	2 per finger	Recognition of simple gestures	a)
4^[^ ^40^ ^]^	Resistive	CNT and textile	Impregnating CNT sheet with the resin	Finger bending degree	Amplitude	2 per finger	Simple Piano skill training	a)
5^[^ ^92^ ^]^	Resistive	Silver nanowire and latex	Dip coating	Pressure of fingertip	Amplitude	1 per finger	Simple grasp object detection	a)
6^[^ ^39^ ^]^	Resistive	PU‐AgNP fiber	Dip coating and pressure‐assisted imprinting	Finger bending degree	Amplitude	1 per finger	Simple VR shooting game	a)
7^[^ ^12^ ^]^	Resistive	Galinstan	Microfludic fabrcation	Palm pressure	Amplitude	3 per finger	Palm pressure mapping	a)
8^[^ ^38^ ^]^	Resistive	Velostat polymer and PDMS	Laser cutting and screen printing	Palm pressure	Machine learning	548 per hand	Gesture of grasp recognition	a)
9^[^ ^80^ ^]^	Triboelectric	Copper and FEP	Depositing and assembly	Finger joint motion angle	Number of peaks	1 per finger	Synchronized robotic hand control	b)
10^[^ ^32^ ^]^	Triboelectric	PEDOT:PSS coated textile	Dip coating	Finger bending degree	Amplitude	2 per finger	Car/drone control	b)
This work	Triboelectric	CNT/TPE coated textile	Spray coating	Finger bending degree	Machine learning	1 per finger	Recognition of complex gestures	b)

^a)^Need external power for sensor operation

^b)^No need to provide power with sensors.

Triboelectric textile glove has been demonstrated in many HMIs to address power consumption and wearability issues.^[^ ^93^ ^]^ However, humidity is considered as a major limitation for TENG due to charge dissipation, decreasing the triboelectric output and inducing a large amplitude variation.^[^ ^94^, ^95^ ^]^ On the other hand, the textile itself commonly exposes to sweat during operation, which may contribute to output deterioration of textile‐based TENGs.^[^ ^96^ ^]^ To ease these issues, M. Zhu et al.^[^ ^1^ ^]^ reported a smart hybrid sock involved piezoelectric sensor as a reference which was not affected by sweat. Recently, endowing textile hydrophobic property is attracting research interest indicated by many works.^[^ ^97^, ^98^, ^99^ ^]^ Until now, researchers normally employ chemical modification,^[^ ^100^, ^101^ ^]^ waterproof package,^[^ ^102^ ^]^ and mimicking hydrophobic microstructure to fulfill the superhydrophobicity requirement.^[^ ^103^, ^104^ ^]^ However, mimicking hydrophobic microstructure may not be suitable for large‐scale fabrication due to the complex manufacturing restriction. Extra packaging does not make the textile itself waterproof but depends on other materials. Hence, a scalable and facile chemical coating method to functionalize textile with hydrophobic property would be desirable since this kind of method is low‐cost and especially making the textile itself hydrophobic.

**Figure 1:**
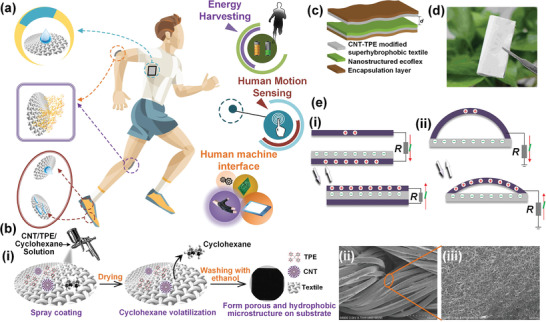
a) Schematic diagram of diversified applications that can be enabled by the developed superhydrophobic triboelectric textile. The man model is reproduced with permission from Freepik.com (https://www.freepik.com/). bi) Fabrication process of the superhydrophobic textile (i.e., positive layer), bii) The SEM image of fibers of superhydrophobic textile and biii) the enlarged view. c) Configuration of the superhydrophobic textile TENG. d) The image of the superhydrophobic textile TENG held by a tweezer, showing its flexibility. e) The triboelectric mechanism of two structures.

Herein, the pristine textile is transformed into superhydrophobic one (**Figure** **1**a) by using a facile, scalable, and cost‐effective coating method. Basically, the superhydrophobic textile is used to scavenge biomechanical energy from human motion as well as to monitor human exercise. Furthermore, using a glove‐based HMI by integrating the superhydrophobic textile, the recognition of complex gestures is achieved by training finger motion signals with machine learning. The accuracy of recognition is just slightly deteriorated from 99.4% to 96.9% in sweat condition owing to the superhydrophobic capability of the device. Finally, 3D VR/AR applications including gun shooting, baseball pitching, and floral arrangement are achieved by gesture recognition based on the glove‐based HMI.

## 2 Hydrophobic Textile Fabrication and Material Optimization

The water contact angle (CA) over 150° is generally used to characterize the superhydrophobic surface which extensively exists in nature such as lotus leaf^[^ ^105^, ^106^ ^]^ and leg of water‐strider.^[^ ^107^ ^]^ Carbon nanotubes (CNTs), intrinsically conductive 1D material, could endow wearable substrate conductivity as well as tailored microstructure. Meanwhile, thermoplastic elastomer (TPE) holds hydrophobic nature and could generate tunable hydrophobic composite microstructure with CNTs,^[^ ^108^ ^]^ which provides a possibility to create a superhydrophobic surface on triboelectric textile for mitigating humidity and sweat issues.

A facile spray coating method is adopted to fabricate a smart superhydrophobic textile for energy harvesting, human motion sensing, and HMIs applications with a minimized sweat effect. As shown in Figure 1bi, the prepared CNTs/TPE solution is spray‐coated onto the pristine polyester textile. After solvent evaporation in the drying process, the CNTs/TPE composite is retained on the textile surface. With a subsequent ethanol etching process, a superhydrophobic textile is obtained. The corresponding SEM image of final superhydrophobic textile is depicted in Figure 1bii and enlarged view of individual fiber surface is shown in Figure 1biii. For negative triboelectrification layer, it is acquired by pouring Ecoflex onto the lotus leaf mold to duplicate the hydrophobic microstructure and then peeling off as shown in Figure S1 in the Supporting Information. Figure 1c presents the assembled textile TENG in which the superhydrophobic textile works as the positive triboelectrification layer as well as the electrodes. The photograph of the assembled TENG that the size of it is 4 cm × 4 cm held by a tweezer is shown in Figure 1d, which indicates its flexible, soft, and thin characteristics that are well fitted with regular clothes for wearable applications. With the simple triboelectric mechanism of two structures given in Figure 1e, the contact‐separation stimulus will induce charges flowing in the external circuit and hence the mechanical energy can be transformed into electricity.

**Figure 2:**
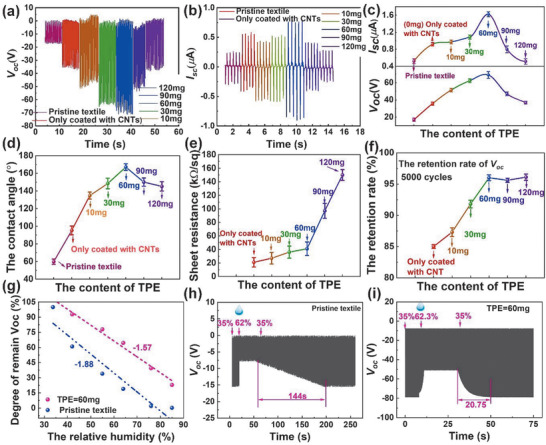
The basic characterization and optimization of TPE content. a) The *V*_oc_, b) *I*_sc_ output, and c) their dependence on TPE content. d) The contact angle, e) sheet resistance, and f) retention rate of *V*_oc_ after 5000 cycles of mechanical loading varies with TPE content. g) The degree of remained *V*_oc_ with increasing RH from 35% to 85% of pristine textile and superhydrophobic textile, indicating the humidity resistance capability of each textile. The real‐time output voltage of textile h) without and i) with superhydrophobic treatment under increased RH from 35% to 62%. (The untreated textile is nonconductive. Therefore, the commercialized conductive textile is attached on the back of it to serve as the electrode.)

Since TPE is of vital importance for the hydrophobic property, we first investigate the appropriate content of TPE in CNTs/TPE solution for a better waterproof performance. The decision of optimized TPE content is made based on several aspects including triboelectric performance, hydrophobicity, conductivity, and mechanical robustness. With an increased TPE content from 0 to 120 mg, **Figure** **2**a–c shows the highest triboelectric open‐circuit voltage *V*_oc_ (72 V) and short circuit current *I*_sc_ (1 µA) in the TPE content of 60 mg. Further increasing TPE content, excessive TPE induced continuous film gradually covers CNTs. Corresponding SEM images (Figure S2, Supporting Information) of increased TPE content clearly show the gradual formation of TPE film and CNTs covering. From the facet of the hydrophobic property, a first CA increase and the following decrease are observed in Figure 2d with the largest CA at 60 mg TPE. With appropriate TPE content (i.e., optimum CNTs/TPE ratio), the hydrophobic composite micro‐/nanostructure may experience most suitable growth surrounding or atmosphere and leading to the largest CA.^[^ ^85^ ^]^ With the help of large CA, the water droplet is difficult to reside at the textile surface as shown in Video S1 (Supporting Information). Considering the superhydrophobic textile serving as electrodes, conductivity investigation under increased TPE content is summarized in Figure 2e. Remarkable rise of sheet resistance can be seen when TPE content is over the 60 mg threshold due to the gradual cover of conductive CNTs by excessive TPE. The slightly decreased CNTs exposure is responsible for the lightly increased sheet resistance before the dramatical resistance increase caused by superfluous insulator TPE. According to the kinetics of materials, the free energy barrier of the formation of a liquid nucleus on surfaces can be expressed as^[^ ^109^ ^]^

(1) $$\begin{array}{c} \Delta G\;=\;\pi \sigma_{ls}\gamma^{*}\frac{\left[2-3\cos\theta +{\left(\cos\theta \right)}^{3}\right]}{3} \end{array}$$

where *σ*_*ls*_ is the liquid‐solid interfacial energy, γ* is the critical radius, and *θ* is the contact angle. To reduce the variables, linear terms *σ*_*ls*_ and γ* are considered as constant for simplification. Thus, the contact angle *θ* makes the difference in the free energy barrier. Meanwhile, the polynomial fitting of the contact angle with the content of TPE is expressed as in terms of the experimental result in Figure 2d

(2) $$\begin{array}{c} \theta =\sum_{i=0}^{4}a_{i}C^{i} \end{array}$$

where *a_i_* is the coefficient, *C* is the content of TPE. Combined (1) with (2), the relationship between free energy barrier and the content of TPE is obtained as shown in Figure S3a in the Supporting Information marked in pink. Higher free energy barrier makes the liquid difficult to nucleus at the superhydrophobic surface leading to less charge dissipation and better TENG performance.^[^ ^103^ ^]^ On the other hand, the sheet resistance of superhydrophobic textiles (Figure 2e) increases as the content of TPE increases, hence the conductivity of prepared textiles decreases (blue line in Figure S3a in the Supporting Information). Higher conductivity contributes to better TENG output. Overall, the two factors free energy barrier and conductivity jointly determine the TENG performance. Hence, there is an optimized content of TPE that shows best TENG performance in the whole material system (Figure S3b, Supporting Information).

Although an increased content of TPE negatively contributes to the conductivity of the electrode in Figure 2e, it serves as the protection layer of CNTs to avoid exfoliation of electrode material caused by repetitive mechanical loading. Thus, Figure 2f shows an increased retention rate of *V*_oc_ with increased TPE content after 5000 cycles loading. The saturation is achieved when the TPE exceeds 60 mg since a good protection has been formed with 60 mg TPE. From all results mentioned above, 60 mg TPE could be an optional choice in terms of desirable triboelectric property, hydrophobicity, conductivity, and durability. Such optimization characterization of TPE content will be the basis of further anti‐humidity capability exploration. Consequently, Figure 2g shows a slower descending in triboelectric output in which the textile is treated with the optimum TPE concentration compared with pristine textile. The superhydrophobic textile possesses better anti‐humidity ability where the degree of *V*_oc_ retention remains at 22.3% in high humidity atmosphere (relative humidity (RH) = 85%) while the output of pristine textile is almost zero in the same condition. The introduction of superhydrophobic surface endowed by composite micro‐/nanostructure relatively mitigates adverse effects on textile TENGs. The mechanism of superhydrophobicity maintaining the triboelectric performance can be expressed from 2 aspects. First, the superhydrophobic interface makes water difficult to generate thin films on the rough micro‐/nanostructure of CNTs/TPE composite (Figure S4a,b, Supporting Information) leading to stronger water repellency as shown in Figure S4c in the Supporting Information.^[^ ^110^ ^]^ Thereby, the adhesion ability of the water droplets on the micro‐/nanostructure is weaker than that of the pristine surface.^[^ ^111^, ^112^ ^]^ Second, the rough surface of CNTs/TPE composite has the advantage of large specific surface area that enhances the triboelectric effect. Furthermore, the superhydrophobic textile can quickly recover from a high humidity environment with a recovery time of 20.75 s which is 7 times shorter than the pristine textile (recover time is 144 s) as shown in Figure 2h,i. When textile TENG escapes from a high humidity atmosphere to the initial environment, quick water molecules desorption from superhydrophobic surface induces quick recovery due to higher energy barrier.^[^ ^108^, ^113^, ^114^ ^]^ Generally, the universal hydrophobic treatment provides a solution to address the issue of triboelectric output susceptible to humidity.

## 3 Biomechanical Energy Harvesting

Realizing energy harvesting from daily wearable garments promotes wearable electronic system integration with the prospect of all‐in‐one e‐textile. Using textiles to scavenge energy from human motion has been extensively investigated in many works. Shen et al. reported a humidity‐resisting TENG for high‐performance biomechanical energy harvesting.^[^ ^115^ ^]^ This work preliminarily investigated the effect of humidity on the performance of proposed hydrophobic TENG. Y. Lai et al.^[^ ^102^ ^]^ used fabric‐based TENG to harvest energy from raindrop, wind and human motion. The waterproof package made fabric‐based TENG insusceptible to water and hence direct energy harvesting from raindrop was realized. However, the fabric itself did not possess waterproof capability but rely on the extra package. Generally, efficient energy harvesting based on textile itself remains a challenge due to the humidity issues, especially in high‐humidity ambient.

**Figure 3:**
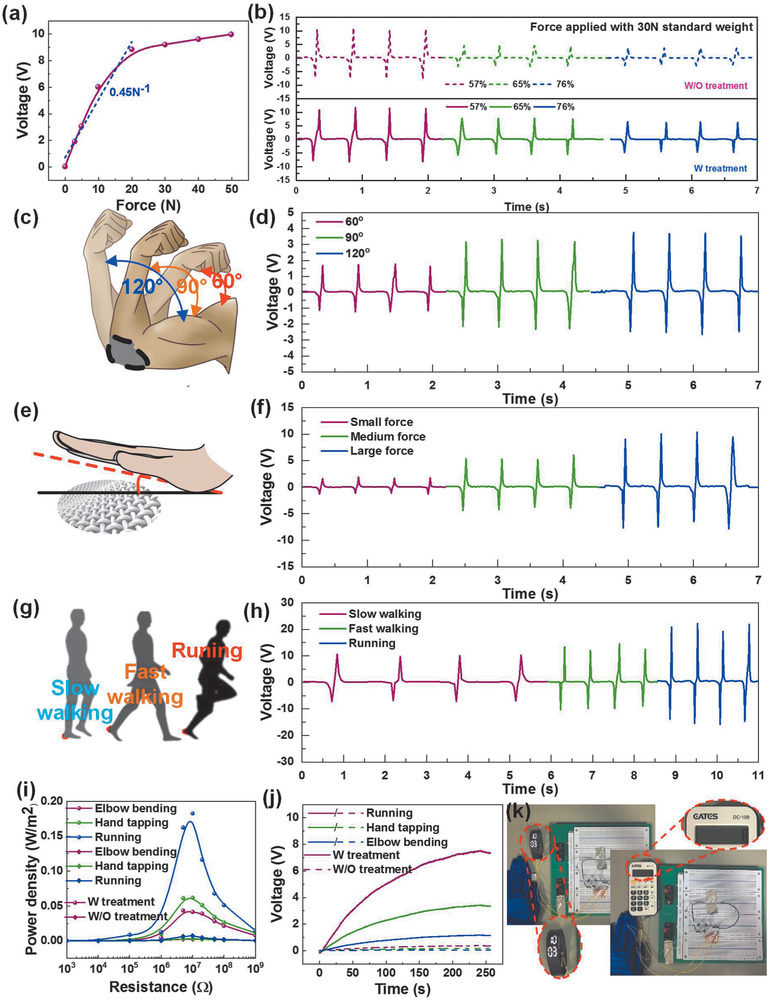
The biomechanical energy harvesting of the superhydrophobic textile TENG. a) Calibration curve of output voltage against force applied by standard weights. b) The real‐time output voltage under increased relative humidity. c) The schematic diagram of device attached on human elbow to harvest the elbow bending energy. d) The output voltage with bending angle of 60°, 90°, 120°. e) The schematic diagram of energy harvesting based on hand tapping, f) the output voltage with small, medium and large force. g) The schematic diagram of energy harvesting based on walking and running. h) The output voltage with slow walking, fast walking and running. i) The power curves of elbow bending, hand tapping, running by using treated textile and untreated textile. j) The charging curves of elbow bending, hand tapping, and running. k) The photographs of powering an electronic watch and calculator using the stored electrical energy in a 10 µF capacitor by biomechanical energy harvesting.

Hence, we use superhydrophobic textiles with the dimension of 8 cm × 8 cm to harvest biomechanical energy from daily human activities. First, the dependence of voltage output on force and humidity are calibrated by standard weights to provide steady and controllable force. As the force increases, the voltage output increases first and then saturates at around 10 V when 30 N is applied. The slope (i.e., sensitivity) of force–voltage calibration curve is 0.4 V N^−1^ (**Figure** **3**a). To demonstrate the humidity‐resistant capability of the proposed superhydrophobic textile, Figure 3b indicates the effect of RH on the voltage output of the pristine textile and the superhydrophobic counterpart. The textile without hydrophobic treatment experiences a dramatic decrease in output as RH goes higher. It almost drops to zero when RH ranges from 57% to 76%. However, superhydrophobic textile TENG remains 50% of its original output even as RH reaches to 76%. Expectedly, the superhydrophobic textile TENG may operate better than the pristine group, especially in the high‐humidity atmosphere. Then the superhydrophobic textile TENG is used to scavenge the energy from elbow bending (Figure 3c), hand tapping (Figure 3e) and walking/running (Figure 3g) in high RH environment (76%) by placing the device at different body parts to verify its efficient energy harvesting capability. Figure 3d shows detailed waveforms for different elbow bending degree of 30°, 60°, and 90°. A larger bending angle makes output increase owing to a larger contact area and force. Similarly, a higher voltage can be seen when the force of hand tapping and walking speed increase as shown in Figure 3f,h. For the power curve measurement, the maximum power density of treated textile (i.e., superhydrophobic textile) is 0.18 W m^−2^ in running case while 0.05 W m^−2^ is for untreated textile (i.e., pristine textile) given in Figure 3i. Meanwhile, capacitor charging curves are presented in Figure 3j. Among three human motions, running generates the largest voltage output as well as the highest charging speed for both treated and untreated textiles. However, charges of pristine textile are taken away by water molecules from the fabric surface at a much faster speed until charges are exhausted while composite micro‐/nanostructure of superhydrophobic textile is beneficial for the rapid desorption of water vapor to prevent deep electrical performance deterioration. Therefore, a more efficient energy harvesting with treated textile is achieved to support the low‐power consumption electronics such as an electronic watch and calculator with 2 min charging as shown in Figure 3k.

## 4 Human Motion Monitoring

Textiles are frequently exposed to sweat in the ambient due to daily wearing. Endowing textile hydrophobic property is required to mitigate the effect of sweat on triboelectric output. Although there are several works that investigated the humidity‐resisting property of hydrophobic textile TENG, the anti‐sweat performance was rarely discussed. Besides, in the case of outdoor activities with garment wearing, physical indexes including motion steps, speed, and burned calories are always interesting parameters for sports‐related applications. Correspondingly, current commercial products such as Mi band, apple watch, and Fitbit are designed to collect such sport indexes. These wearable electronics, however, are mainly based on rigid semiconductor fabrication techniques and not conformable with the curvature of the human body. Hence, a hydrophobically functionalized textile with good conformability is prospective to realize stable and anti‐interfered human motion sensing.

**Figure 4:**
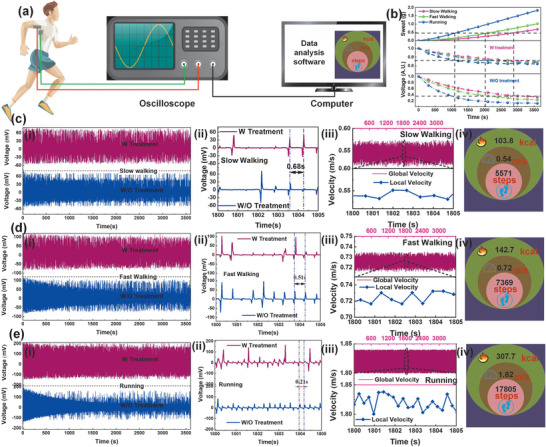
The human exercise monitoring. a) The schematic diagram of human exercise monitoring system with the calculation capability of moving steps, velocity, distance, and burned calories. The man model is reproduced with permission from Freepik.com (https://www.freepik.com/). b) The calibration curves of output voltage on sweat volume with slowing walking, fast walking, and running based on treated textile and untreated textile. The output voltage of 1 h slowing walking fast walking and running with treated (red line) and untreated textile (blue line). The c–ei) output voltage, c–eii) time interval, c–eiii) instantaneous velocity, and c–eiv) the display interface of calculated steps, distance, and burned calories with c) slow walking, d) fast walking, and e) running.

Firstly, the anti‐sweat capability of proposed superhydrophobic textile is investigated. The device with the size of 6 cm × 6 cm is sewed onto the garment underneath the armpit. One layer locates at underarm while another layer is sewed onto the cloth at the chest side as shown in **Figure** **4**a. The friction between two triboelectrification layers could generate voltage peaks which are in different time intervals when people swing their arms in different motions such as walking, fast walking and running. Under the condition of 1 h exercise, the simulated sweating curves are depicted in the first column of Figure 4b, and detailed measurement and calibration can be found in the Experimental Section. Artificial sweat is sprayed to superhydrophobic textile every 5 min in 1 h measurement. As shown in the second column in Figure 4b, the superhydrophobic textile TENG experiences a much slower output descending as sweat volume increases. Nearly 85% of output remains even after 1 h slow walking while the output retention rate of untreated textile TENG dramatically decreases to around 30% due to its hygroscopic nature as shown in the third column of Figure 4b. Figure 4ci presents the voltage output of real 1 h slow walking of a female adult. The treated textile, slightly decreasing to 92% of maximum output, is superior to untreated textile since its output is reduced to 34%. Similarly, the output retention rate of treated textile remains 84% and 80% in fast walking and running cases respectively as shown in Figure 4di and Figure 4ei. Secondly, inspired by commercial smartwatch or wrist band with the function of measuring motion steps, speed and burned calories, we demonstrate these functions of our wearable superhydrophobic TENG. From the enlarged view (Figure 4cii) of Figure 4ci, the time interval of two positive/negative peaks can be extracted by the algorithm and hence the corresponding real‐time motion speed can be read with a known step distance 37 cm as shown in Figure 4ciii. Besides, motion steps are obtained by counting output peaks. By involving the empirical formula of burned calorie calculation,^[^ ^116^ ^]^ burned calories are also displayed in the MATLAB interface as shown in Figure 4civ. Similarly, cases of fast walking and running are demonstrated in Figure 4d,e, respectively. The result indicates that proposed superhydrophobic textile TENG has a good anti‐sweat performance which is highly desirable for human motion monitoring without obvious output degradation in the sweating circumstance, especially in the condition of strenuous exercise.

## 5 Gesture Recognition in VR/AR Applications

**Figure 5:**
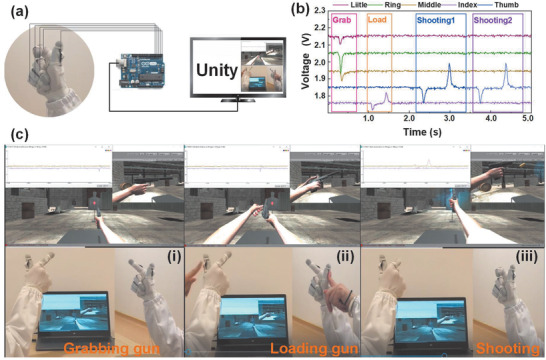
The demonstration of shooting game, which is based on the amplitude of output signals. a) The schematic diagram of the control system. b) Signal patterns of grabbing, loading gun, and shooting. c) The corresponding screenshot of grabbing, loading gun, and shooting in VR space of Unity.

The glove is a frequently used wearable textile item in daily life. The low‐cost glove‐based HMI equipped with triboelectric sensors can fulfill requirements of self‐powered control.^[^ ^80^ ^]^ Here, we fabricate a glove‐based HMI with the superhydrophobic textile TENG sensors distributed on the individual fingers of the glove to demonstrate a VR shooting game control. The sensors on gloves are in single‐electrode mode if there is no special statement (Figure S5, Supporting Information). Besides, the dependence of voltage output on finger bending degree is depicted in Figure S6 in the Supporting Information. As shown in a schematic diagram of control in **Figure** **5**a, each sensor channel is connected to Arduino for data acquisition with the sensor response time ≈100 ms (Figure S7 and Table S1, Supporting Information). Through serial port control, Python can process acquired data in a real‐time manner and send a command to Unity based on TCP/IP communication. A shooting game control, including grabbing the gun, loading the gun and shooting, is achieved by 3 distinct signal patterns. First, middle, ring, and little fingers bend to make superhydrophobic textile contact with Ecoflex, which is defined as grabbing the gun with three negative peaks in the signal pattern (Figure 5b). The virtual hand in Unity responds to the corresponding order and grabs the gun as shown in Figure 5ci. In the second stage, the sensor in the thumb is excited by left‐hand press to trigger loading action as shown in Figure 5cii. Finally, index bends for shooting. The corresponding signal and screenshot are presented in Figure 5b and Figure 5ciii, respectively. A detailed video demonstration can be found in Video S2 (Supporting information). By such a demonstration, it is proved that the simple gesture recognition can be a choice for two or more states control.

**Figure 6:**
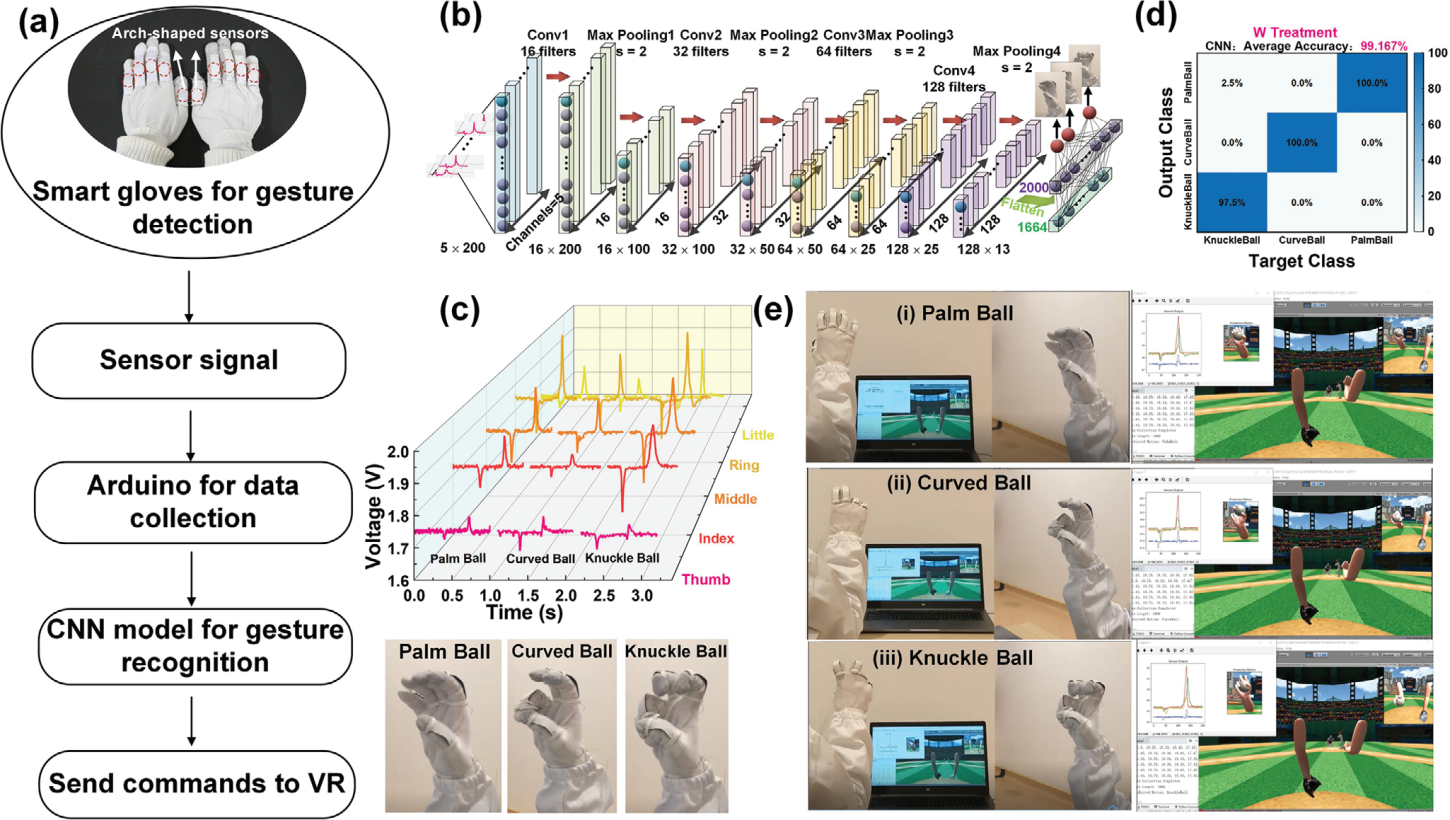
The demonstration of baseball game scenario with machine learning. a) The flow chart for gesture recognition and control. b) The structure of CNN model. c) The signal patterns of 3 gestures. d) The confusion matrix for 3 common gestures of pitching ball. e) The photographs of 3 gestures (left), and corresponding screenshot of using gestures to achieve VR control in Unity (right).

Triboelectric signals have much subtle information that cannot be distinguished by naked eyes or be simply differentiated by signal amplitude or peak number. As an emerging technique for extracting subtle differences, machine learning has been used in triboelectric signal pattern analysis.^[^ ^71^ ^]^ Triboelectric signals from human fingers contain abundant hints of many similar and complex gestures where the difference in the appearance of the signal is hardly distinguished in a manual method. Thus, we demonstrate a VR baseball scenario based on 3 similar pitching ball gestures recognition. As the flow chart shows in **Figure** **6**a, the triboelectric signal from glove will be obtained by Arduino MEGA 2560 with 8 integrated circuits of amplifier. The convolutional neural network (CNN) in Python will recognize gestures and give a corresponding order to Unity through TCP/IP communication. The process and parameters for constructing the CNN model can be found in Table S2 in the Supporting Information and Figure 6b. The signal from each sensor is recorded with 200 data points (5 sensors in total) and 200 samples are collected for each gesture where 120 samples used for training (60%), 40 samples used for validation (20%), and 40 samples used for testing (20%). As can be seen, signal patterns for 3 gestures of throwing the ball, including palm ball, curved ball and knuckle ball, are very similar in terms of signal appearance (Figure 6c). After the training process in the CNN model, a high recognition accuracy 99.167% is achieved as depicted in Figure 6d. After 50 training epochs, the accuracy is almost 98.3% as shown in Figure S8 in the Supporting Information. Such a high accuracy provides a great potential for real‐time control based on gesture recognition of similar signal patterns through machine learning. Figure 6ei–iii show screenshots of using 3 pitching ball gestures to control the virtual hand in Unity. Detailed Video S3 in the Supporting Information can be found in supplementary materials. This demonstration shows the feasibility of using machine learning to achieve highly accurate gesture recognition with similar signal patterns and real‐time control in virtual space.

**Figure 7:**
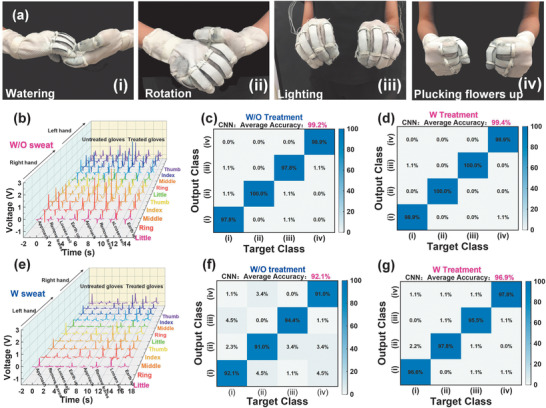
The illustration of superhydrophobic glove for better accuracy under sweat condition of wearer. a) Photographs of four gestures with similar signal patterns. The signal patterns of untreated and treated glove b) without sweat and e) with sweat. Without sweat, the confusion matrix for c) untreated glove and d) treated glove. With sweat, the confusion matrix for f) untreated glove and g) treated glove, showing that the glove with superhydrophobic textile maintaining a high accuracy even in a sweating condition.

The abovementioned gestures use less than 10 fingers. Furthermore, recognition of more complex gestures involving 10 fingers is demonstrated to study the universality of the glove‐based HMI for various gesture recognition. It aims at building a universal interface or platform for various gestures, in which gestures involve either 10 fingers or less than 10 can be applicable. To first verify the advantage of the superhydrophobic glove, the accuracy for gesture recognition based on gloves with treated and untreated sensors is compared when sweat appears. In the training sample collection process, each of 10 sensors has 200 data points recorded per action to train the model for recognition. For each gesture, 400 samples are collected for training (60%), validation (20%) and testing (20%). Here same CNN structure is used as shown in Figure 6b except the input size becomes 10*200 (10 sensors, 200 data points for each sensor) and the final output size becomes 4 for four gestures. In **Figure** **7**a, four gestures using 10 fingers are respectively defined as ‘watering,’ ‘rotation,’ ‘lighting,’ and ‘plucking’ for following flower arrangement in AR space. In the condition without sweat, signal patterns of these four gestures from untreated and treated gloves are presented in Figure 7b. Each gesture employs signals from all fingers and occupies 10 channels. A high accuracy (around 99%) of recognition for both groups is achieved as shown in Figure 7c,d. However, under sweat condition (sweat weight: 0.43 g), the untreated glove experiences a dramatic decrease in the signal amplitude, leading to a signal appearance change (Figure 7e) and consequently lowering the accuracy to 92.1% (Figure 7f). The treated group remains 45% of the original output (Figure 7e) and desirable accuracy (96.9%, Figure 7g). Generally, the untreated group exposes to large amplitude decrease, inducing signal amplitude almost compromised to zero. This changes the overall shape of signal patterns and reduces the occupied signal channel number. The optimized CNN model cannot recognize anymore unless parameters for CNN are reoptimized with newly collected data samples under specific sweat conditions. By contrast, the superhydrophobicity of treated group maintains the output at a recognizable level in the sweating atmosphere. It helps to avoid the endless effort of collecting a large quantity of data samples under different sweat conditions.

**Figure 8:**
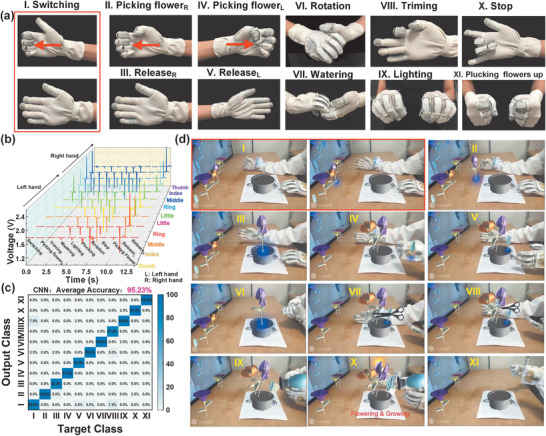
The AR demonstration of flower arrangement based on complex gesture recognition using machine learning. a) The photographs of eleven gestures. b) Signal patterns of eleven gestures. c) The confusion matrix of gesture recognition. d) The corresponding screenshot of eleven gestures in AR space of Unity.

After the compared trial of untreated and treated gloves, the benefit of superhydrophobicity is indicated. A flower arrangement in AR space is conducted based on four aforementioned gestures plus another seven gestures. Except ‘watering,’ ‘rotation,’ ‘lighting,’ and ‘plucking,’ another seven gestures involving less than 10 fingers are added for interesting AR flower arrangement application including ‘switching,’ ‘picking flower (right hand),’ ‘release (right hand),’ ‘picking flower (left hand),’ ‘release (left hand),’ ‘triming,’ and ‘stop’. Photographs of these eleven gestures are shown in **Figure** **8**a. Their corresponding signals are summarized in Figure 8b. Here, 200 data samples of each gesture are obtained for training (60%), validation (20%) and testing (20%). After the training process in the CNN model, average accuracy for gesture recognition can reach up to 95.23% as depicted in the confusion matrix of Figure 8c. Based on such highly accurate recognition, a real‐time AR flower arrangement is demonstrated by wearing the glove‐based HMI. Next, the procedures of arranging flowers are described. As shown in Figure 8d, first, users wear gloves to switch in the AR space to choose a wanted flower and then pick the flower to flowerpot with rotating to appropriate visual angle (Figure 8dI–VI). Three flowers are picked for demonstration (repeating the steps in Figure 8dI–VI for three times). Following the leaf trimming process, watering and light exposure are performed to make flowers bloom and grow (Figure 8dVII–X). A ‘stop’ signal is used for terminating the watering and lighting. Finally, all the flowers are plucked up (Figure 8dXI). When the user makes these gestures, virtual flowers in the AR space will be controlled to perform corresponding actions. It should be noted that the signal of ‘switching’ is composed of negative peaks for contact and positive peaks for separation, and the successful recognition depends on the whole contact‐separation cycle. Comparably, the signals of other gestures only consist of negative peaks or positive peaks. Thus, in Figure 8dI, there are two images for ‘switching’ to show the intact gesture while one image is for other gestures as shown in Figure 8dII–XI. Meanwhile, the detailed AR demonstration is shown in Video S4 (Supporting Information).

In order to clearly identity the uniqueness of this novel glove‐based HMI, the previous works are summarized in Table 1. Finger bending degree, pressure and strain are popular and interesting parameters for human hand related motion monitoring. First, among the listed 3 mechanisms commonly used for the flexible/stretchable electronics development, the triboelectric effect is a desirable solution for the realization of self‐powered sensors while another 2 mechanisms require external power supply. Second, most of reported works realize gesture recognition based on the difference of signal amplitude and number of peaks that may contain limited information and cannot help achieving the recognition of complex gestures without the assistant of advanced analysis tool (e.g., Machine learning). Third, in general, large number of sensors could provide more abundant and accurate information to achieve comprehensive gesture recognition. But this approach increases the complexity of fabrication and data processing. Overall, with the help of machine learning as glove can perceive complex gestures with minimal number of self‐powered sensors for the first time. Moving forward, glove‐based HMI combined with machine learning, such a universal platform, has the prospect to achieve more complex and comprehensive control by involving more gestures.

## 6 Conclusion

The polyester textile is converted into a superhydrophobic one for energy harvesting, human motion sensing, and self‐powered HMI with minimized sweat or humidity effect by using a facile and cost‐effective CNTs/TPE coating manufacturing method. The superhydrophobic textile experiences a quick recovery from a high‐humidity environment with the recovery time of 20.75 s which is 7 times shorter than the pristine textile (144 s) as well as threefold boosted triboelectric performance. The power density of superhydrophobic triboelectric textile to scavenge biomechanical energy from human activities (0.18 W m^−2^) is 4 times higher than that of pristine textile (0.05 W m^−2^) in high‐humidity atmosphere. With anti‐sweat capability, the superhydrophobic textile enables human exercise monitoring without obvious output voltage deterioration. The voltage output remains 80% with minimized sweat effect even after 1 h exercise. Furthermore, a, low‐cost, self‐powered, intuitive glove‐based HMI with proposed superhydrophobic triboelectric textile sensors is developed. By leveraging machine learning, a minimalist design with each finger containing only one triboelectric sensor can perform recognition of complex and similar gestures by using glove‐based HMI. Benefited by the superhydrophobic characteristic, the negative effect of sweat is minimized, leading to an improved recognition accuracy (96.7%) compared to that without superhydrophobicity (92.1%). Finally, 3D VR/AR controls including shooting game, baseball pitching, and floral arrangement are successfully achieved using the developed glove interface, showing its great potential in diversified VR/AR applications. Looking forward, the combination of machine learning with low‐cost, self‐powered and minimalist‐designed gloves explores a new possibility of a universal platform for various recognition tasks of complex gestures.

## 7 Experimental Section

##### Preparation of CNTs/TPE Solution and Spray Coating

60 mg CNTs was fully dispersed in 200 mL cyclohexane followed by 2 h ultrasonication. The content of CNTs was fixed at 60 mg since it was used as electrode material. Then different contents of TPE (0/10/30/60/90/120 mg) was dissolved in prepared CNTs suspension with 2 h ultrasonication. In this way, a series of CNTs/TPE suspensions with different TPE content were obtained for optimization purpose. The spray coating process onto textiles was executed by the spray coater (USI, PRISM 400). The air pressure for spraying was maintained at 45 psi, the distance between the spray‐gun and the substrates was≈10 cm, and the spraying speed was ≈ 0.5 cm s^−1^ to steadily control the spray coating process. Then samples are immersed in the ethanol solution (purity: 99%) to etch the surface for 5 min.

##### The Fabrication of Assembled Superhydrophobic Textile TENG

The superhydrophobic textile was used as positive triboelectrification layer as well as the electrode. The pristine textile was used to encapsulate the triboelectric layer. The microstructure Ecoflex was attached on superhydrophobic textile electrode to fabricate negative layer. The encapsulation layer was also pristine textile. Finally, these two layers were assembled together to form a narrow‐gap textile TENG

##### Calibration and Measurement of Output Curve Under Simulated Sweat Condition

As a study shows,^[^ ^117^ ^]^ an adult was at exercise with a sweat rate of about 0.24 to 0.464 mg cm^−2^ min^−1^. 0.24, 0.352, and 0.464 mg cm^−2^ min^−1^ are chosen for sweating simulation of slow walking, fast walking and running respectively. Since efficient contact area of device with human body here was around 48 cm^2^, the amount of generated sweat at specific device area ranges from 691 to 1336.32 mg in 1 h exercise. Artificial sweat was purchased and contained in 15, 20, and 30 mL spray bottles, and each spray delivers 0.058 g, 0.084 g, and 0.11 g solution, respectively. The bottle sprayed once every 5 min.

##### The Fabrication of Smart Glove

Ecoflex is coated on fingers of gloves with drying 30 min in 80 °C oven. Then the superhydrophobic textile with encapsulation layer was on the back of glove fingers in arch‐shaped structure. The work mode of arch‐shaped triboelectric sensors on gloves was in single electrode mode. For the motivation of using sing electrode mode in glove design, first, the two‐electrode mode of sensors on glove will generate 20 electrodes for 10 fingers in total that increases the fabrication complexity of glove and wearing burden due to increased conductive wires. Second, in terms of sensors output amplitude of single‐electrode mode, it was effective for the analysis of machine learning, which was indicated by the successful recognition of complex gesture in single‐electrode mode.

##### Measurements

The photos are taken by a digital camera (Canon EOS 70D). The SEM images are acquired from Hitachi S‐4800 cold field emission SEM at an accelerating voltage of 5 kV. Open‐circuit voltage and short‐circuit current measurement are performed by a Keithley Electrometer (Model 6514). Other voltage measurements are carried out by a DPO‐5034B oscilloscope (Tektronix) with the normal 10 MΩ probe. A multifunction digital four‐probe tester (JG, ST‐2258C) was used to measure the sheet resistance of samples.

##### Study Participation

Prior to participation in the experiments, informed consent was obtained from the volunteer in all experiments.

## Conflict of Interest

The authors declare no conflict of interest.

## Supporting information

Supporting Information

Supplemental Video 1

Supplemental Video 2

Supplemental Video 3

Supplemental Video 4
