# Supplementary material for: Machine Learning Glove Using Self‐Powered Conductive Superhydrophobic Triboelectric Textile for Gesture Recognition in VR/AR Applications
Source: Adv Sci (Weinh). 2020 Jun 9;7(14):2000261. doi: 10.1002/advs.202000261 (PMC7375248; doi:10.1002/advs.202000261)
Supplement: Supplementary file 1 — Supporting Information [file ADVS-7-2000261-s001.pdf]

Supporting Information

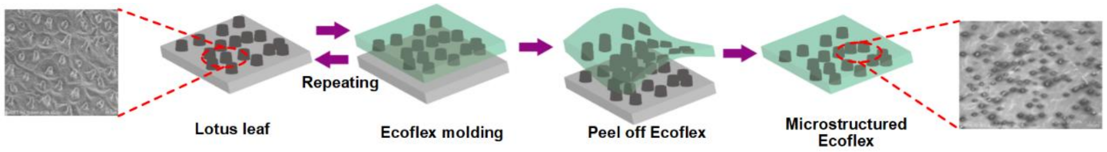

Figure S1. The schematic diagram of using ecoflex to replicate complimentary superhydrophobic micro-/nanostructure of lotus leaf.

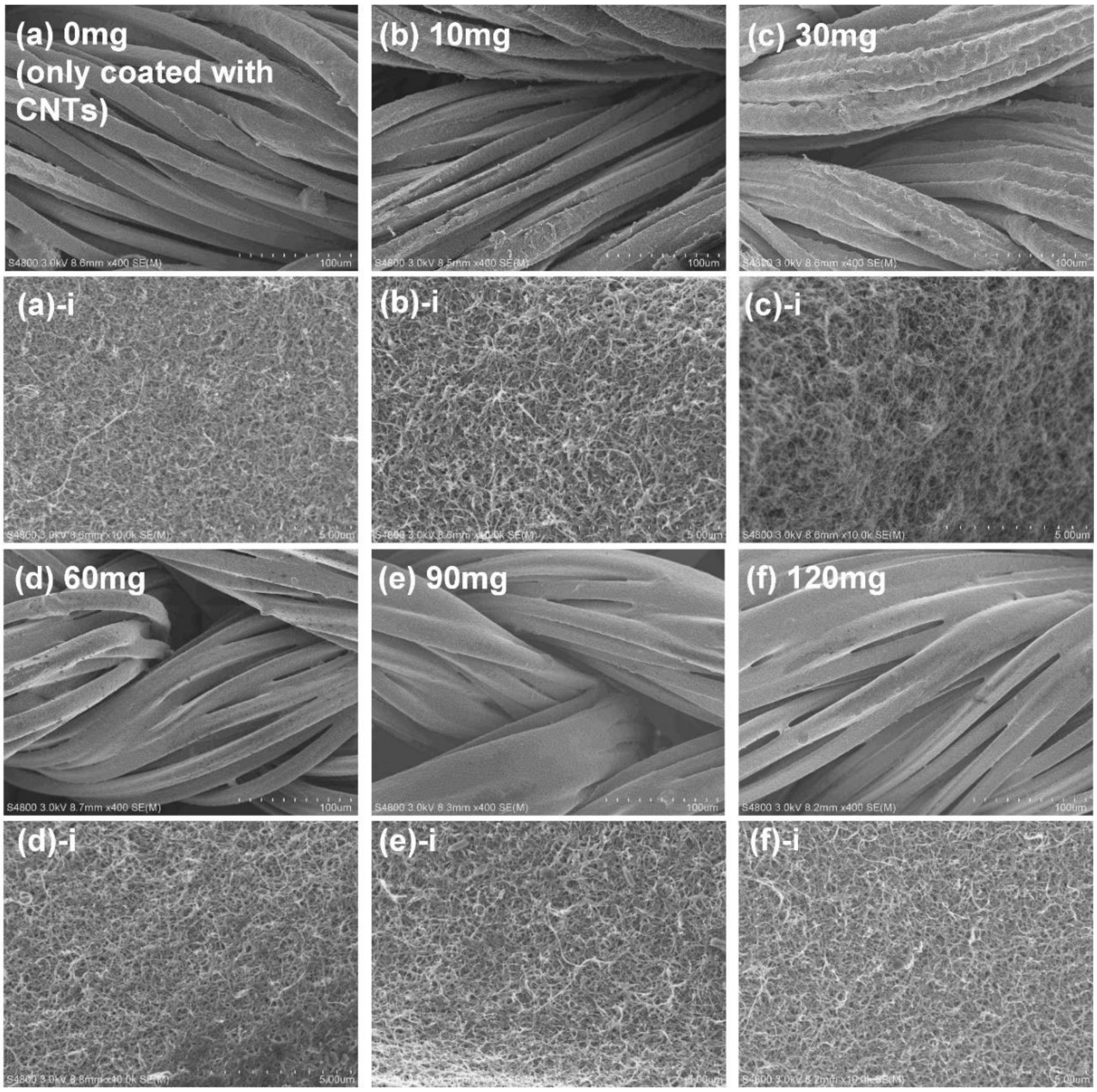

Figure S2. SEM images and their enlarged view at different TPE content. (a)(a-i) Only coated with CNTs (i.e. 0mg), (b)(b-i) 10mg, (c)(c-i) 30mg, (d)(d-i) 60mg, (e)(e-i) 90mg, (f)(f-i) 120mg.

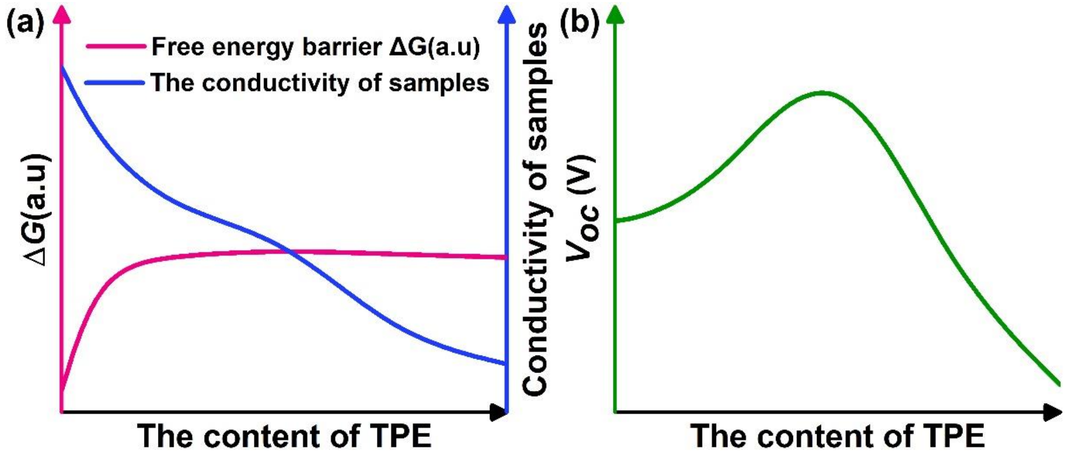

Figure S3. (a) the theoretical changing curve of the free energy barrier, the conductivity, and (b) the voltage output of the textile TENG with the increase of the content of TPE.

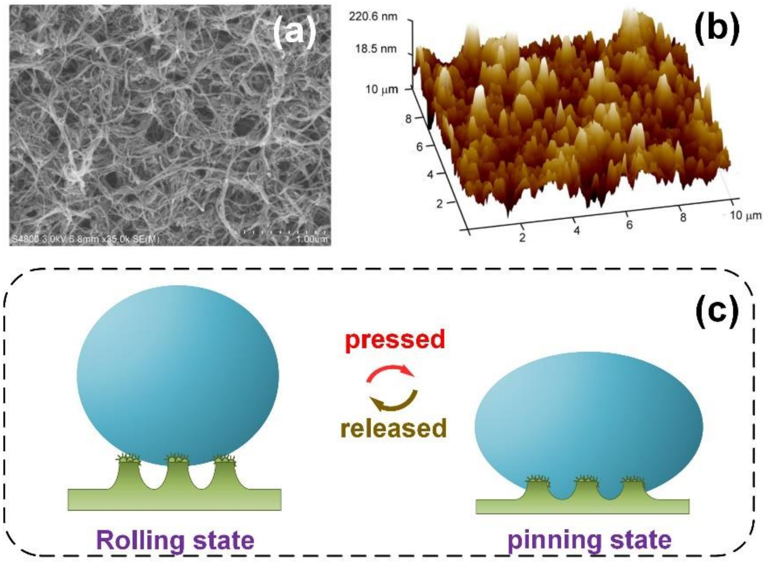

Figure S4. (a) The SEM image shows the rough microstructure of CNT/TPE treated textile. (b). The 3D AFM image showing the surface height variation of a piece of as-prepared CNT/TPE treated textile. (c) The water repellency model.

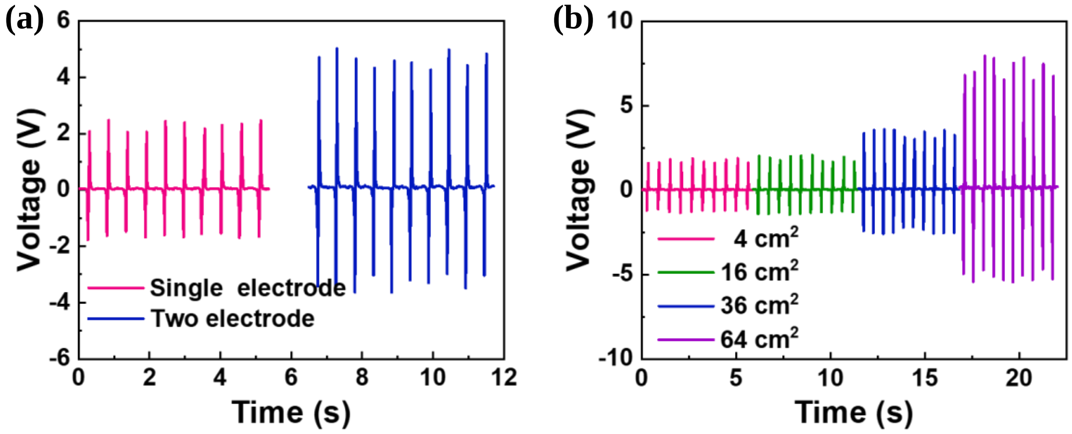

Figure S5. (a) The output comparison of single-electrode and two-electrode modes. (b) The dependence of output on area.

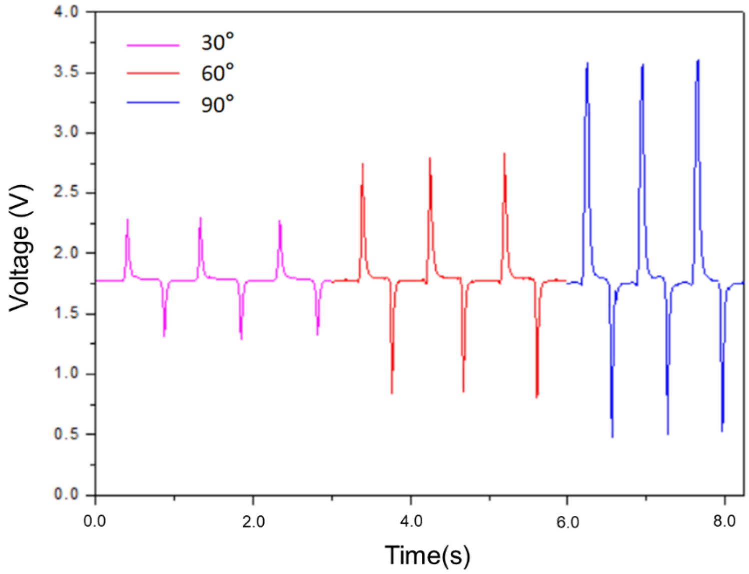

Figure S6. The dependence of voltage output on the finger bending degree.

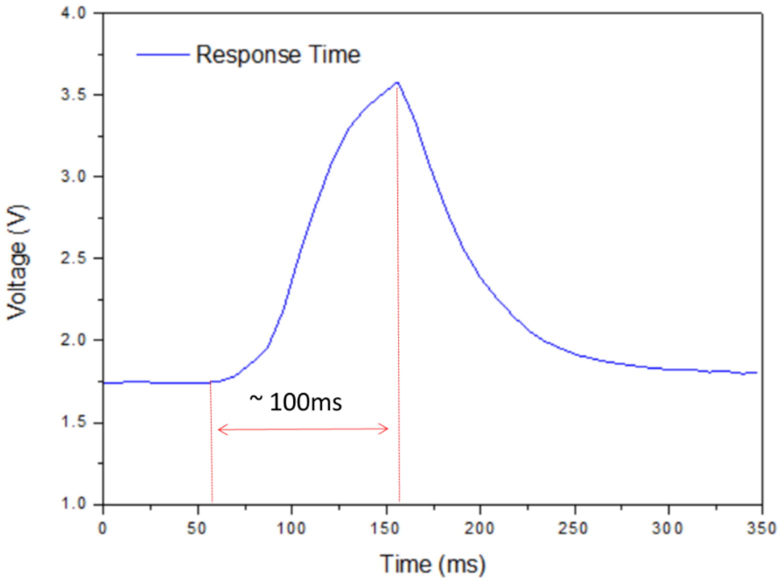

Figure S7. The response time of the triboelectric finger sensor.

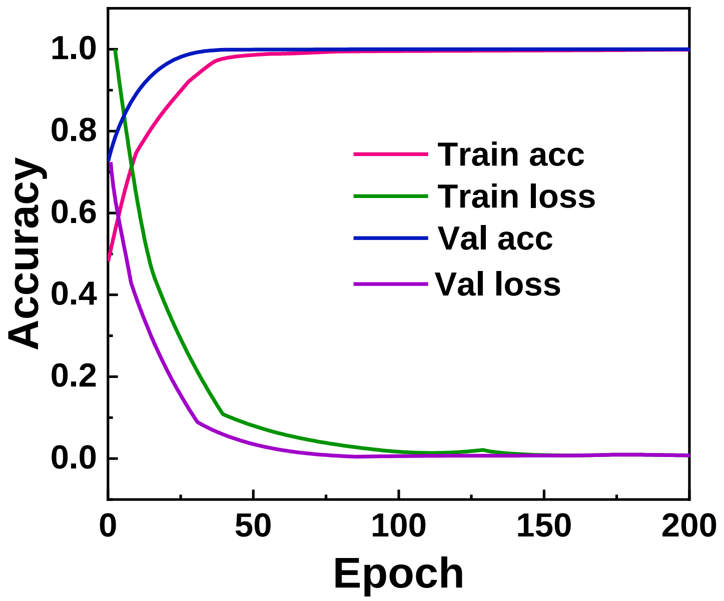

Figure S8. Accuracy variation with respect to the training epoch.

Table S1. The value of response time of 10 times measurements.

No.	1	2	3	4	5	6	7	8	9	10	Average
Response time (ms)	104.2	112.8	112.8	104.2	95.6	95.6	104.2	86.8	95.6	112.8	102.5

Table S2. The parameters for constructing Convolutional Neural Network (CNN)

No	Layer Type	No. of Filters	Kernel/ Pool Size	Stride	Input Size	Output Size	Padding
1	Convolution 1	16	5	1	(None, 200,5)	(None, 200, 16)	same
2	Max Pooling 1		2	2	(None, 200,16)	(None, 100, 16)	same
3	Convolution 2	32	5	1	(None, 100, 16)	(None, 100,32)	same
4	Max Pooling 2		2	2	(None, 100,32)	(None, 50,32)	same
5	Convolution 3	64	5	1	(None, 50,32)	(None, 50,64)	same
6	Max Pooling 3		2	2	(None, 50,64)	(None, 25,64)	same
7	Convolution 4	128	5	1	(None, 25,64)	(None, 25,128)	same
8	Max Pooling 4		2	2	(None, 25,128)	(None, 13,128)	same
9	Flatten				(None, 13,128)	(None, 1664)	same
10	Dense (2000)				(None, 1664)	(None, 2000)	
11	Dense (3)				(None, 2000)	(None, 3)	
